# Toward a study of culture suitable for (*Frontiers* in) cultural psychology

**DOI:** 10.3389/fpsyg.2013.00392

**Published:** 2013-07-02

**Authors:** Tuğçe Kurtiş, Glenn Adams

**Affiliations:** Cultural Psychology Research Group, Department of Psychology, University of KansasLawrence, KS, USA

“The mutual constitution of culture and mind is a topic of central importance across all of areas of psychology.” With this mission statement, *Frontiers in Cultural Psychology* declares *mutual constitution*—the process by which mind and culture “live together, require each other, and dynamically, dialectically, and jointly make each other up” (Shweder, 1990; p.1)—as the unifying theme of its otherwise diverse contributions. Implicit in this theme is a non-essentialist understanding of both culture and mind. One direction of this dynamic process refers to the *cultural constitution of mind.* Mind is not simply an expression of genetic blueprint; instead, it emerges through *active participation* in and *ongoing engagement* with sociocultural affordances present in the structure of everyday worlds^1^. The other direction of this dynamic process refers to the psychological constitution of cultural worlds. Cultural worlds and their particular affordances do not exist apart from human activity; instead, they persist (or not) because people in the ongoing flow of everyday life actively select and (re)produce features—and de-select others—that resonate with their particular beliefs and desires. Among other examples, this dynamic conception of culture and mind is evident in work that considers the mutual constitution of moral understandings and sleeping practices (Shweder et al., 1995); motivational orientations and constructions of success and failure situations (Kitayama et al., 1997); affective orientations and children's books (Tsai et al., 2007); personal identity and societal master narratives (Hammack, 2011); or national identity and constructions of history (Carretero, 2011).

Despite this non-essentialist vision, much of the work that carries the label of cultural psychology continues to reflect and reproduce problematic reifications of culture and self (see Hermans and Kempen, 1998; Adams and Markus, 2004; Gjerde, 2004; Hammack, 2011). One could identify many examples to illustrate this point. The particular case that provoked the present commentary is an article by Mojaverian et al. (2013).

The strength of the article lies in the productive use of comparison across cultural settings to illuminate the cultural-ecological foundations of professional help-seeking. Conventional scientific wisdom portrays as standard (and prescribes as optimal) models of professional help that emphasize explicit socio-emotional support and patient disclosure as part of the treatment process. The comparison across settings suggests that these models do not operate as some context-general law; instead, they reflect context-specific dynamics of self and relationship that are prevalent within the European American settings that disproportionately inform scientific imagination. The same models may be ineffective or counterproductive (e.g., by inflicting additional stress) when imposed without reflection upon other settings. By illuminating the cultural-ecological foundations that underlie standard models of professional help, this comparison across cultural settings directs attention to the historical processes that produce “normal” standards, helps to de-naturalize conventional scientific wisdom (see Adams et al., 2012), and contributes to the anti-essentialist vision of cultural psychology implicit in the mutual constitution framework.

Problems of reification arise—in both the article and the field as a whole—in associations of *culture* with essentialist categorical distinctions. Researchers rarely provide context-sensitive accounts of cultural-ecological patterns or affordances, and instead rely on broad national or racial/ethnic categories as problematic shorthand for cultural engagement or participation. There could be defensible reasons for the authors' citation of patterns observed among “Asians,” “Asian Americans,” “Chinese Americans,” “East Asians” as the basis for hypotheses about (or interpreting results of) Japanese participants. However, the re-deployment of these categories without a discussion of particular cultural-ecological patterns—and the corresponding implication that US-born, English-speaking students of Asian descent have more in common with Japanese students than with their “Caucasian” American colleagues—appears to locate the source of commonality in some shared, racial group essence.

Similar issues are evident in conceptions of *cultural competence* (see Kirmayer, 2012 for a review on, critiques of, and conceptual alternatives to cultural competence), which the article associates with “creation of ethnic-specific mental health services [that] focus on incorporating cultural values … and encouraging cultural understanding between mental health practitioners and their clients” (p.7). Too often, such “cultural understanding” amounts to little more than racialized stereotypes of otherness that reproduce and homogenize across dubious categories (see Mudimbe, 1988, on “invention of Africa” or Lewis and Wigen, 1997 on the “myth of continents”). These practices do damage not only because they exaggerate and falsely fix boundaries between people in ways that are artificial and absolute (see Lutz and Abu-Lughod, 1990; Kirmayer, 2012), but also because they reflect and reproduce long-standing systems of racist and imperialist oppression (e.g., see Shaw, 2005; Kirmayer, 2011; see also Said, 1978, on Orientialism).

Regrettably, such entity-based conceptions of culture and unconsidered use of problematic categories have become standard practice, even within otherwise critical perspectives of cultural psychology. Despite aspirations to challenge generalizations about humanity, these practices promote the un-reflexive (and often unwitting) reproduction of stereotypical generalizations about “cultural differences” and “cultural others.” Despite aspirations to illuminate the dynamic fluidity of human psychological experience, these practices portray the cultural constitution of self (an emerging process) as the production of different varieties of self (as a more-or-less finished product). In other words, these practices promote not only reification of culture, but also reification of self.

As an antidote to problematic reifications of culture and self, the mutual constitution framework emphasizes the ongoing, dynamic production of culture and mind. From this perspective, cultural participation is less about conscious indoctrination into bounded systems of timeless traditional values than it is engagement with particular cultural-ecological patterns: that is, the structures of everyday worlds—including institutions, practices, artifacts, and discursive tools—that scaffold psychological experience Kroeber and Kluckhohn (1952). At the same time, cultural worlds are not static, timeless entities, but reflect and require culturally grounded actors who continually reproduce them with the psychological charge of their particular desires and beliefs. Rather than confine people in invented traditions (e.g., Hobsbawm and Ranger, 1983) of imagined racial communities (e.g., Anderson, 1983), this articulation provides a more productive conception of culture adequate for *Frontiers in Cultural Psychology*. This conception promotes “cultural understanding” not by rehearsing problematic ethnic stereotypes, but instead by revealing the broader historical processes at work in the production of “normal” scientific standards.

## Footnotes

^1^Our references to *affordances* emphasize the points of contact between a non-reifying cultural psychology and various articulations of “ecological” psychology (Barker, 1968; Gibson, 1979; see Oishi and Graham, 2010). Besides providing tools for a more dynamic approach to the mutual constitution theme, ecological orientations to cultural psychology also make contact with sociocultural-historical versions of the Cultural Psychology project (e.g., Cole, 1996).
